# Synovial Macrophages: Past Life, Current Situation, and Application in Inflammatory Arthritis

**DOI:** 10.3389/fimmu.2022.905356

**Published:** 2022-07-26

**Authors:** Lin-Kun Bai, Ya-Zhen Su, Xue-Xue Wang, Bing Bai, Cheng-Qiang Zhang, Li-Yun Zhang, Gai-Lian Zhang

**Affiliations:** ^1^ Third Hospital of Shanxi Medical University, Shanxi Bethune Hospital, Shanxi Academy of Medical Sciences, Tongji Shanxi Hospital, Taiyuan, Shanxi, China; ^2^ First Affiliated Hospital of Dalian Medical University, Dalian Medical University, Dalian, China; ^3^ Fifth Hospital of Shanxi Medical University, Shanxi Provincial People’s Hospital, Taiyuan, Shanxi, China

**Keywords:** arthritis, synovial membrane, synovial macrophages, macrophages, cell subsets, treatment

## Abstract

Inflammatory arthritis is an inflammatory disease that involves the joints and surrounding tissues. Synovial hyperplasia often presents when joints become inflamed due to immune cell infiltration. Synovial membrane is an important as well as a highly specific component of the joint, and its lesions can lead to degeneration of the joint surface, causing pain and joint disability or affecting the patients’ quality of life in severe cases. Synovial macrophages (SMs) are one of the cellular components of the synovial membrane, which not only retain the function of macrophages to engulf foreign bodies in the joint cavity, but also interact with synovial fibroblasts (SFs), T cells, B cells, and other inflammatory cells to promote the production of a variety of pro-inflammatory cytokines and chemokines, such as TNF-α, IL-1β, IL-8, and IL-6, which are involved in the pathogenic process of inflammatory arthritis. SMs from different tissue sources have differently differentiated potentials and functional expressions. This article provides a summary on studies pertaining to SMs in inflammatory arthritis, and explores their role in its treatment, in order to highlight novel treatment modalities for the disease.

## Introduction

Joint injury plays an important role in inflammatory arthritis. Chronic inflammation involving bone tissue is known to play a role in the destruction of bones, which is mainly accomplished through the action of osteoclasts (OCs). However, the small number and short survival time of normal human OCs, which are difficult to isolate from bone, have set back investigations that study bone destruction mechanisms in joints (1). Recently, almost all chronic arthritic lesions have been shown to have inflammation of the mesenchymal tissue, including synovium, tendons, ligaments, and joint capsules, while damage to bone and cartilage alone has been shown to take place in a few exceptional cases (2). Accordingly, these findings have led to speculation that perhaps cells other than OCs are involved in the development of bone destruction. Patients with rheumatoid arthritis (RA) have significant proliferation of synovial tissue, and their synovial lesions can induce further joint destruction (3). Therefore, synovial lesions in arthritis are currently being widely and thoroughly studied.

**Figure 1:**
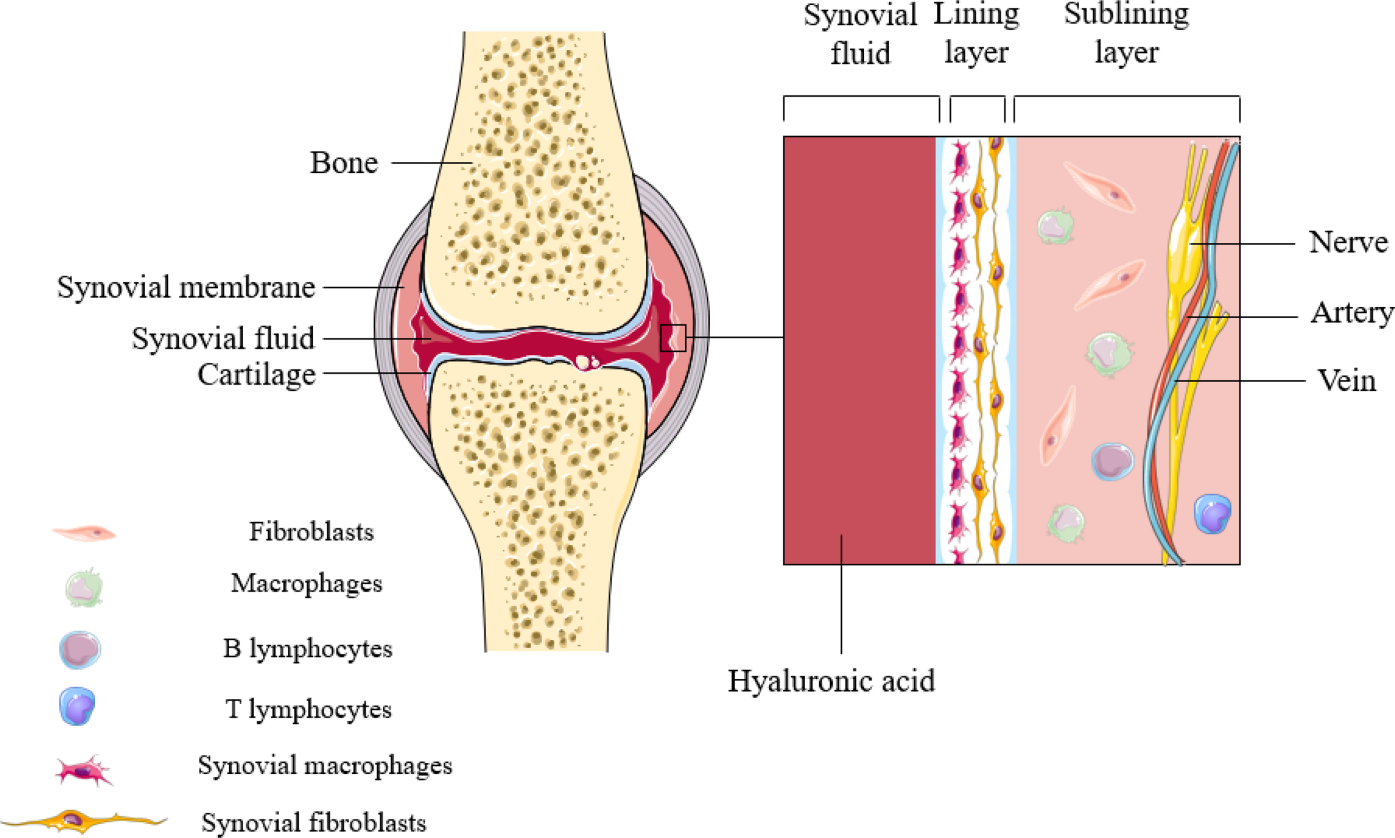
Synovial structures in inflammatory arthritis. Synovial macrophages are mainly distributed in the lining layer of synovial tissue. During the onset of the disease, SMs can not only induce inflammation, but also perform immune monitoring.

The synovium is a highly specialized mesenchymal tissue with a lining and sub-lining surrounding the joint (**Figure 1**). The thinner but highly cellular layer of lining consists of two main cell types: SFs and SMs. SFs provide the extracellular matrix (ECM) that supports synovial structures and secretes hyaluronic acid and lubricin in order to maintain synovial fluid function. Unlike SFs, however, SMs extend pseudopods into the synovial space so as to maintain intra-articular homeostasis. The supporting sublayer contains a rich network of lax connective tissue, underlying fibroblasts and macrophages, sympathetic and sensory nerves, and blood and lymphatic vessels that provide oxygen and nutrients. Other immune cells (lymphocytes, mast cells, and dendritic cells) are seldom found in normal synovium, and are mainly distributed in the perivascular area of the sublayer, playing a role in immune drainage (4). Populations of SMs have been reported to persist in the lining layer in the vast majority of patients with arthritis who responded adequately to treatment, and these macrophage subsets may be resident sentinels involved in maintaining tissue homeostasis (5).

Factors such as cell developmental origin (embryonic and adult bone marrow-derived cells), organ survival environment, microbial invasion, tissue damage, and activation or inactivation signals from other immune cells determine the differentiation of the various subsets of macrophages (6). SMs, as a subtype of tissue-resident macrophages (TRMs), maintain tissue homeostasis, and play a key role in controlling infection as well as excessive inflammation, similar to other cell subtypes (7–18) (**Table 1**). Resting SMs are round or oval in shape and are surrounded by radial protrusions; the filamentous pseudopods elongate to make the cells irregular in the functionally active state. Activated SMs both induce inflammation and carry out immune monitoring *via* multiple pattern recognition receptors (19). One particular study divided SMs into two subpopulations according to the expression of the chemokine receptor CX3CR1: CX3CR1^+^ lining macrophages and CX3CR1^-^interstitial macrophages (20). In mice, CX3CR1^+^ macrophages have been shown to form a protective and tightly connected cell layer that prevents arthritis *via* isolation of the synovium while preventing the infiltration of inflammatory cells (21). Similarly, TREM2^+^ trigger receptors and tight junction genes associated with their barrier function, which are highly expressed on human SMs, have been described to protect joints while maintaining the homeostasis of the intra-articular environment (22). In light of the aforementioned findings, an increasing number of researchers have started to focus on the therapeutic potential as well as the specific immune mechanisms of SMs to treat a variety of diseases both *in vivo* and *in vitro*. This paper reviews the latest studies on the individual development and biological functions of SMs and highlights prospects for the applications of SMs in a variety of inflammatory arthritis conditions.

**Table 1:** Markers and functions of tissue-resident macrophages (TRMs).

TRMs	Tissue	Function	Induced activator	Reference
Adipose tissue macrophages	Fats	Regulates insulin sensitivity, adaptive thermogenesis	Metabolic stimulation (free fatty acids, high insulin, high sugar)	Russo et al. (7) Caslin et al. (8)
Alveolar macrophages	Lung	Initiation of pulmonary immunity, pulmonary immune monitoring, maintenance of tissue homeostasis	Bacterial lipopolysaccharides, hyperoxic partial pressure, surfactants, signals provided by alveolar type I and type II cells	Dewhurst et al. (9) Joshi et al. (10)
Lung interstitial macrophages	Lung	Regulates DC maturation and activation, antigen presentation	Inhaled granules, bleomycin, radiation	Dewhurst et al. (9) Shi et al. (11)
Bone marrow macrophages	Marrow	Mobilize hematopoietic stem cells to support hematopoiesis	Elevated hemoglobin levels	Heideveld and van den Akker et al. (12)
Intestinal macrophages	Gastrointestinal tract	Maintains homeostasis in the intestinal environment, activates antigen presentation of T cells, a high phagocytic capacity	Gut microbes	Bain and Schridde et al. (13)
Microglia	CNS	Promote neuronal survival, participation in immune detection and synaptic remodeling	Foreign antigens (bacteria, fungi, parasites, and viruses), brain damage	Nayak et al. (14)
Kupffer cells	Liver	Remove microorganisms and cellular debris from the blood, produce a variety of inflammatory cytokines and proteases	Bacterial endotoxins, liver damage (alcohol, fat)	Basit et al. (15)
Red marrow macrophages	Spleen	Red blood cell clearance, iron metabolism, reticulocyte quality control	Elevated hemoglobin levels	Heideveld and van den Akker et al. (12) Hashimoto (16)
Synovial macrophages	Synovial membrane	M1: Recruit inflammatory cells, cause joint erosion M2: Promote angiogenesis, tissue reconstruction and repair	IFN-γ, LPS, GM-CSF IL-4, IL-13, M-CSF	Zhang et al. (17) Teng et al. (18)

## Development of SMs

### Discovery and Origin of SMs

Takasugi and Hollingsworth (23) were the first to discover a large group of phagocytes in the synovial fluid of RA patients in 1967, which was not studied in depth due to the limitations of experimental techniques during that time. With the development of minimally invasive joint surgery and ways in obtaining synovial tissue, this group of cells has been found to be macrophage-like cells, or SMs, which are one of the two main cell types that make up the arthritic synovium. Subsequently, high-throughput techniques found that the SM population of arthritis patients in clinical remission can be stably present within the lining layer, while stimulated SMs can induce and exacerbate inflammation by activating inflammatory mediators in the local microenvironment. Such technologies have broadened the understanding of disease heterogeneity and pathophysiology, opening up more avenues for discovering SMs as new potential therapeutic targets (24).

SMs, as a type of macrophage, have long been thought to be derived primarily from monocytes differentiated from hematopoietic stem cells (HSCs) in bone marrow. Until the end of the 20th century, many TRMs were understood to proliferate independently of the bone marrow hematopoietic system, with a considerable number of tissue macrophages being derived from primitive macrophages present in the yolk sac or fetal liver. Embryonic yolk sacs can directly produce embryonic macrophages at about the age of the embryo (E) 7.5 and spread throughout the blood circulation in embryo at approximately E9.0, undergoing symbiotic differentiation into TRMs (25). Following the widespread use of gene sequencing technology, Bian (26) et al. found that yolk sac-derived myeloid-biased progenitors (YSMPs) can migrate to the fetal liver after E11.5, producing lineages such as monocytes, which subsequently migrate into tissues. Monocytes begin to differentiate into TRMs before birth and exhibit different phenotypes depending on the tissue in which they are located. Unlike infiltrating macrophages derived from bone marrow HSCs, TRMs are able to sense tissue damage, participate in the inflammatory response, and constantly repair tissue homeostasis. In order to further identify the cytological characteristics of bone marrow-derived synovial macrophages (BMSMs) and embryonic synovial macrophages (ESMs), SMs of different origins were then identified (21, 27). Accordingly, most ESMs were shown to be major histocompatibility complex class II negative (MHCII^-^) in the joint synovium. Moreover, the depletion of ESMs was shown to worsen inflammation, indicating that it plays a major role in maintaining tissue integrity and limiting inflammation. Unlike ESMs, the BMSMs’ effect of MHCII^+^ is transient, and when inflammation occurs, BMSMs continuously replenished by monocytes play a primary role in maintaining the number of SMs during the inflammatory phase. Meanwhile, ESMs only exhibit low levels of local proliferation, allowing their protective signals to be masked by a relatively large number of BMSMs. Under steady-state conditions, however, BMSMs have not been shown to contribute to the number of SMs. During the same period, Tu Jiajie et al. (28) used CX3CR1^+^ cells expressing green fluorescent protein in CX3CR1^+^/GFP mice in order to track the appearance of SMs at different stages of the embryo and bone marrow chimeras in adult mice. In doing so, ESMs appeared in the mid-embryonic stage, manifested as F4/80^+^CD11b^-^, exploded after birth, and expressed anti-inflammatory cytokines such as IL-4 and IL-10. Meanwhile, BMSMs appeared in the late embryonic stage and manifested as F4/80^-^CD11b^+^, while cell number increased during progression of the disease in adult mice alongside a reduction in the regression period, suggesting that BMSMs had certain pathogenic effects.

### Typing of SMs

Recently, the function of different subtypes of SMs and their regulators has garnered increased attention. Numerous studies on single-cell sequencing techniques have been conducted to explore the heterogeneity of SMs in arthritis patients, which has ushered in unique perspectives for discovering potentially new therapeutic targets in inflammatory arthritis (19). Fan Zhang et al. (29) performed multimodal transcriptional plus proteome mapping analysis on the synovial tissue of 36 patients with RA, producing a high-dimensional single-cell dataset of synovial tissue in RA patients. Here, M1 SMs and M2 SMs were shown to be the two extremes of the continuous spectrum of activated monocytes in inflammatory synovial tissue of patients with RA, with characteristics similar to macrophages, as shown in **Table 2** (30–32). M1 SMs cause joint destruction and erosion through the secretion of cytokines such as TNF-α, IL-1β, IL-12, and IL-18 (17) and are closely associated with synovial inflammation in inflammatory arthritis. M2 SMs can be further divided into M2a, M2b, M2c, and M2d, which promote angiogenesis, tissue remodeling, and repair by producing a large number of anti-inflammatory factors including IL-10, TGF-β, and arginase1 (33). M0 (quiescent phase) SMs can transform into M1 (pro-inflammatory) or M2 (anti-inflammatory) SMs when stimulated differently, which may be regulated by a variety of factors, including JAK/STAT, PI3K/Akt, JNK, Notch/NF-κB, and B7-H3/STAT3 signal pathways (34). M1 SMs have also been shown to transform into M2 SMs. Semaphorin-3A (SEMA-3A), which is derived from bone marrow stromal cells (18), lactic acid (35), and acupuncture at the foot San Li point (36), can accelerate this process.

**Table 2:** Characteristics of M1 macrophages and M2 macrophages.

Characteristic	M1	M2
Inducer	IFN-γ, LPS, GM-CSF	IL-4, IL-13, M-CSF, helminth
Marker	NOS2, TLR2, TLR4, CD80, CD86, CX3CR1	CD115, CD206, PPARG, ARG1, CD163, CD301, Dectin-1, PDL2, Fizz1, CX3CR1
Secreted cytokines	IL-12, IL-23, TNF-α, IL-1β, IL-8, IL-6	IL-10, IL-4, IL-13, TGF-β
Secreted chemokines	CXCL9, CXCL10, CXCL11, CCL5	CCL17, CCL22
Correlated transcription factors and signal regulators	STAT1, IRF5, NF-κB, SOCS1	STAT6, IRF4, SOCS3, KLF4, PPARP-γ, c-Myc
Surface receptors	MHC-II	CD206, mannose, MGL, STAB1, CD163
MicroRNA	miRNA-29, miRNA-33, miRNA-127, miRNA-155	miRNA-146a, miRNA-222, miRNA-223, let-7c
Function	Recruit inflammatory cells, cause joint erosion, promote inflammation	Promote angiogenesis, promote tissue reconstruction and repair, anti-inflammatory, promote tumor growth and invasion

Interestingly, the Accelerating Medicine Partnership consortium combined single-cell RNA sequencing (scRNA-seq), mass spectrometer, batch RNA-seq, and flow cytometry in order to analyze 51 synovial tissue samples from patients with RA and osteoarthritis (OA), providing the first comprehensive description of 18 unique synovial cell populations (29). Unlike classical typing of SMs as described above, the authors divided mononuclear-macrophage subsets into SC-M1 (IL-1B^+^), SC-M2 (NUPR1^+^), SC-M3 (C1QA^+^), and SC-M4 (IFN^+^). Accordingly, the amplification of TLR-activated pro-inflammatory SC-M1 and SC-M4 with interferon (IFN) characteristics were observed to be more pronounced in the synovium of patients with RA compared to those with OA. The corresponding findings were also consistent with recent comparative analyses of synovial tissue transcriptome profiles and reference transcriptomes of immune cells activated by endogenous and exogenous stimulants in RA patients along with OA patients (37). Subsequently, Kuo and his team (38) conducted a study using a similar approach. They found that heparin-binding EGF-like growth factor (HBEGF)-positive clusters 1 and 4 macrophages acted similarly to SC-M1 and SC-M4, and that the relative abundance in RA was higher than that in OA. Thus, the transcriptional profiles of SC-M2 and SC-M3 were shown to not be clearly defined by specific markers, indicating that they differed from known activated macrophage states, which suggested that these two subtypes may be steady-state macrophage phenotypes. Overall, the complex heterogeneity of SMs reflects health status as well as disease at different stages, which provides a better understanding regarding the biological functions of macrophages while laying the foundation for treating the different subsets of SMs.

## Markers of SMs

Markers of SMs refer to important indicators that define and sort all SMs during development, which assisted in the identification of different subpopulations of SMs in human and animal synovial tissues.

### Pro-Inflammatory Surface Markers: CD80, CD86, and Ly6C

CD80 and CD86 are co-stimulating molecules on the surface of macrophages that can act as biomarkers in predicting pro-inflammatory SMs. Anti-CD80 and anti-CD86 therapy in CIA mice have been shown to significantly inhibit disease scoring and morbidity (56). Liu et al. (57) utilized immunohistochemistry to detect the synovial tissue in 18 patients with RA, demonstrating that CD86^+^ macrophages were present in 11 of them and were surrounded by lymphocyte aggregation. In addition, the stimulation of resident macrophages *in vitro* with *Bacillus thuringiensis* Cry1Ac protoxin (pCry1Ac) has been shown to upregulate the expression of CD80 and CD86, thereby enhancing the production of pro-inflammatory cytokines TNF-α, IL-6, and MCP-1 (58). When CD80 and CD86 that are expressed in SMs bind to the shared receptor CD28, CD4^+^ T cells can be co-stimulated, thus activating pro-inflammatory signaling pathways and increasing the production of IL-2 and IFN-γ (59). Moreover, the CD80/CD86 axis has been shown to play an important role in the pro-inflammatory process of SMs, though it cannot be used as a specific marker for M1-type SMs. In addition, researchers (60) have detected the presence of the marker Ly6C in mouse synovial tissue BMSMs of E20.5 *via* immunohistochemistry and flow cytometry, which serves as a marker for mouse circulating monocyte–macrophage lineages. Ly6C^high^ monocytes in the mouse joint synovium are known to be involved in the development of arthritis, while Ly6C^low^ monocytes help reduce joint inflammation by mobilizing Treg cells (61). Cremers et al. (62) injected collagenase into the joint cavity of wild-type C57BL/6 mice to induce local arthritis symptoms, which exhibited a strong increase in S100A8/A9 expression during the advanced stage of inflammation as well as an increase in the number of Ly6C^high^ monocytes flowing into the synovium. The corresponding findings suggested that the development of synovitis may be mediated by Ly6C^high^ monocytes–macrophages, and the molecular marker S100A8/A9, which occurs during inflammation, is also involved in this process. However, during the same period, Misharin et al. (63) found that non-classical Ly6C^-^CD62L^-^CD43^+^CCR2^-^ monocytes initially differentiated into M1 macrophages so as to drive inflammatory arthritis in mice, which then polarized to M2 macrophages as inflammation progressed. In contrast, Ly6C^-^ has been shown to act as a polarizing marker for M2 SMs in arthritis and mediate the reduction of joint inflammation at the onset of disease. This suggests that Ly6C plays a different role in monocyte-derived macrophages and TRM, contrary to the findings described above. Whether this discrepancy is related to the difference between classical and non-classical, and whether Ly6C can be used as a specific marker for M1 type SMs, should be further validated in large cohorts studies of patients and animal models with inflammatory arthritis.

### Anti-Inflammatory Surface Markers: CD163, CD206, and F4/80

The haptoglobin–hemoglobin receptor CD163 and mannose receptor CD206 have been described to be highly expressed in chronic arthritis M2c and M2a macrophages, respectively (64). Compared with wild-type (C57BL/6), CD163^-/-^CIA mice have been observed to have higher arthritis scores, earlier onset, longer disease, and intense progression (65). Meanwhile, CD163^-/-^CIA mice mainly exhibit the Th2 response, while CD163^+/+^CIA mice mainly undergo Th1 reactions. Baeten et al. (66) performed macrophage and lymphocyte subset analysis on synovial biopsy samples from 26 patients with spondylitis (SpA) as well as 23 patients with RA, which demonstrated a significant increase in CD163^+^ SMs that was associated with systemic inflammation and impaired T-cell activation. The above findings show that CD163 has a novel strong anti-inflammatory effect, and may complement the anti-inflammatory T-cell effect. Similarly, CD206 may also play an important role in the anti-inflammatory ability of SMs. Yokozeki et al. (67) injected TGF-β inhibitor (SB431542) intraperitoneally into C57BL/6J mice and used real-time PCR to detect the expression of CD206 in intervertebral disc macrophages, in which the proportion of CD206^+^ macrophages was found to be significantly reduced. At the same time, CD206^+^ macrophages have also been shown to regulate the IL-6-mediated paracrine mechanism in order to combat fibrosis in fibroblasts (68). Therefore, it may be reasonable to posit that CD163 and CD206 serve as markers of anti-inflammatory SMs. Guo Yawei et al. (60) found that F4/80 and CD11b can dynamically monitor mouse ESMs and BMSMs through immunohistochemistry and flow cytometry separation. Here, only F4/80^+^ESMs were found in the mouse synovial tissue of E12.5, while CD11b^+^BMSMs appeared in mouse synovial tissue of E20.5. The synovial tissue of newborn to adult mice was found to be a mainly mixed cell population of ESMs and BMSMs, while the proportion was more ambiguous, suggesting that the expression and function of SMs from two different sources differed but overlapped. Dexmedetomidine (DEX) is a highly selective alpha2-adrenoceptor agonist that is known to increase the expression of F4/80^+^Ly6G^+^ macrophages, further triggering the secretion of TGF-β1 and leading to inhibition of cytokine storms and accelerated inflammation resolution (69). F4/80^+^ cells have been shown to have good immunotherapy potential in inflammatory arthritis, and the cellular-FLICE inhibitory protein (c-FLIP, Flip) has been described to serve as a regulator of RA synovial F4/80^hi^ SMs. Huang et al. (70) induced Flip^f/f^LysM^c/+^ mice with a mild inflammatory phenotype and found that, on day 9, following the induction of arthritis, the number of F4/80^hi^ SMs in the joint synovium of Flip^f/f^LysM^c/+^ mice increased while that of Flip decreased. Meanwhile, F4/80^hi^ SMs were shown to possess an anti-inflammatory phenotype in both Flip^f/f^LysM^c/+^ and control mice. These findings may have been because a decrease in Flip is known to alter intracellular signaling, thereby promoting a rise in the number of F4/80^hi^ SMs with an M2-like phenotype, though it cannot rule out the influence of other cells. Subsequently, researchers have found that bone marrow-derived M1 macrophages could express markers of M2 macrophages after GM-CSF stimulation *in vitro*, suggesting that using only specific markers on the cell surface to distinguish between M1 SMs and M2 SMs may not achieve the desired effect (71).

### Imaging Markers: Folate Receptors β and Transporters

Folate receptor β (FRβ) is a glycosylphosphatidyl (GPI)-anchored plasma membrane protein that is expressed on activated SMs. In light of its strong affinity for folic acid, this receptor is an important SM imaging marker and RA therapeutic target (e.g., folate conjugate PET tracers and folate conjugate drugs) (72). FRβ has been found to be expressed at higher levels in SMs that are polarized towards anti-inflammatory and repair aspects, a property similar to CD163 (73). Samaniego et al. (74) proposed that FRβ has now been used as a target for imaging as well as the delivery of therapeutic agents in inflammatory arthritis. Therefore, FRβ may also be useful in delivering agents with the ability to alter the polarization state of macrophages. Translocator protein (TSPO) is a high-affinity cholesterol and drug-binding protein that is highly expressed in SMs (CD163^+^ and CD68^+^) and activated synovial stroma in patients with RA (75). Gent et al. (76) used (R)-[¹¹C] PK11195-based positron emission tomography (PET) to target TSPO on activated SMs and image subclinical arthritis to provide the possibility of early diagnosis and disease-sensitive surveillance. Therefore, TSPO can also be used as an imaging marker for arthritis SMs. As understanding of the pathogenesis of SMs continues to develop, such imaging markers may provide new targets for the future treatment of inflammatory arthritis.

### Other Markers

Other markers such as CD32, CD64, CD68, MerTK, and CX3CR1 also play important roles in SM definition and sorting. Using immunofluorescence staining technology, researchers have found that multiple markers exist simultaneously in synovial tissue. Specifically, the inner membrane lining layer mainly contains the markers CD163, CD32, and CD68, while the lower synovial layer has CD68, CD163, CD32, and CD64, of which CD163 and CD68 can identify SMs in the late maturation stage of RA patients (33). Manferdini et al. (77) analyzed synovial tissue in patients with first-generation (P1) and fifth-generation (P5) OA *via* flow cytometry and found that the typical markers CD14, CD16, CD68, CD80, and CD163 of SMs in P1 isolated synovial cells were positively expressed, while P5 synovial cells only had positive labels for SFs. Meanwhile, MerTK^+^CD206^+^ macrophages in the synovium of patients in sustained remission were found to be significantly increased compared to patients in active or intermittent remission of arthritis, which was inversely correlated with disease activity, synovial hypertrophy, and angiogenesis (22). The corresponding finding suggests that MerTK and CD206 play a synergistic role in the anti-inflammatory process of SMs. Culemann et al. (21) found that in CX3CR1^+^/GFP mice, the synovial lining layer macrophages selectively express the markers CX3CR1, CD68, and F4/80 under steady-state conditions, accounting for 40% of the total number of SMs. In contrast, macrophages within the synovial stromal have not been shown to express CX3CR1. IL-6, TNF-α, and CCL5 secreted by M1 SMs, along with IL-10, TGF-β, and CXCL13 secreted by M2 SMs, are all related to the pathology of inflammatory arthritis. Therefore, these cytokines and chemokines can also act as secretory markers, which may assist in distinguishing between SMs subtypes of different functions. In regard to current research, the specific markers of SMs remain to be fully understood; hence, finding substances that can specifically label SMs has become an immediate issue.

## Interaction of SMs With Other Immune Cells

**Figure 2:**
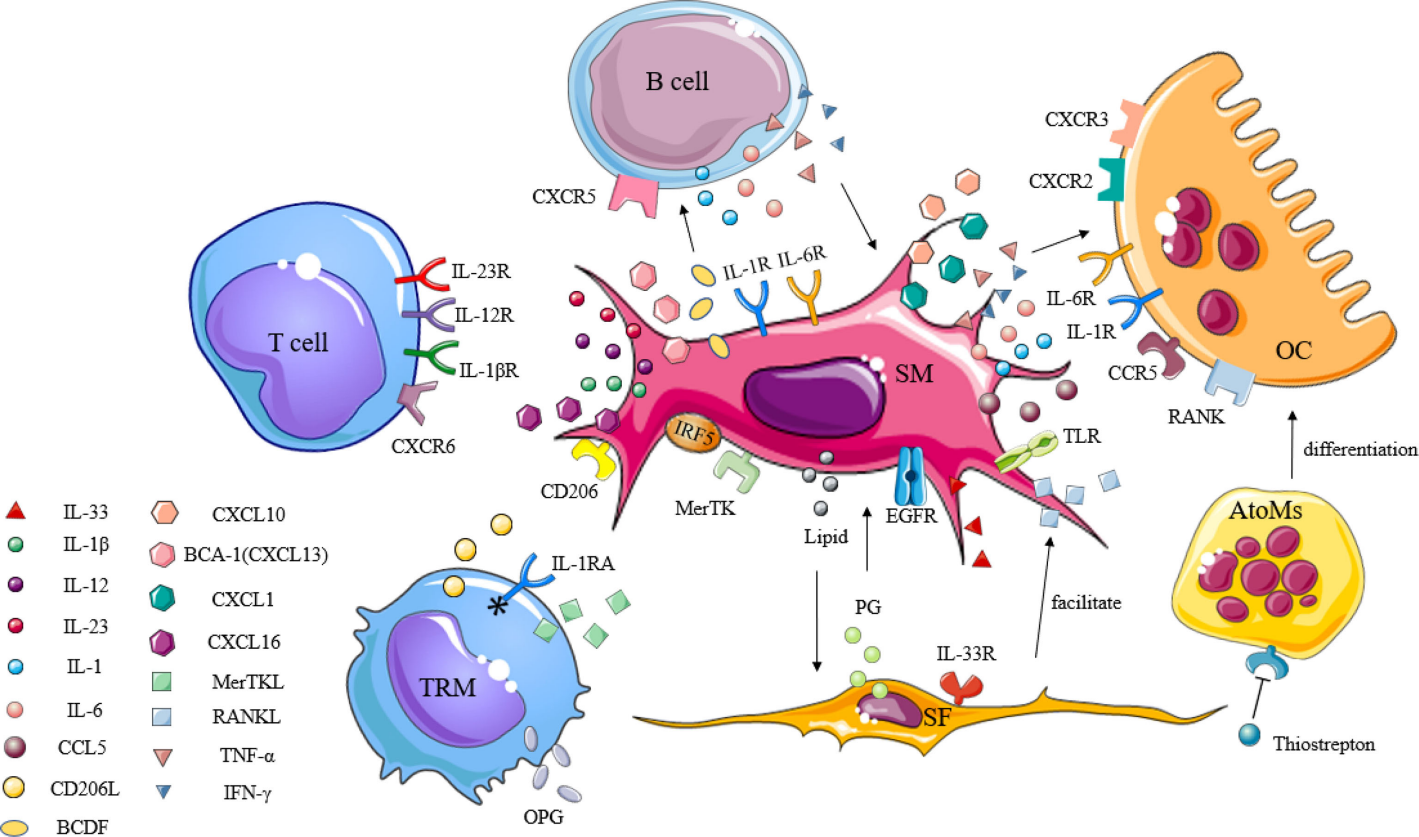
The immunogenic role of synovial macrophages in inflammatory arthritis. Synovial macrophages interact with other immune cells through cytokines, chemokines, and inflammatory mediators to promote the activation, proliferation, and differentiation of lymphocytes, synovial fibroblasts, and osteoclasts in the synovium. TRM, tissue-resident memory T; SM, synovial macrophage; SF, synovial fibroblast; OC, osteoclast; AtoMs, arthritis-associated osteoclastogenic macrophages; IL-1RA, IL-1 receptor antagonists; IL-1βR, IL-1β receptor; IL-12R, IL-12 receptor; IL-23R, IL-23 receptor; IL-1R, IL-1 receptor; IL-6R, IL-6 receptor; IL-33R, IL-33 receptor; CD206L, CD206 ligand; CXCL, C-X-C motif chemokine ligand; CXCR, C-X-C motif chemokine receptor; CCL, C-C motif chemokine ligand; CCR, C-C motif chemokine receptor; TNF-α, tumor necrosis factor-α; IFN-γ, interferon-γ; OPG, osteoprotegerin; MerTK, tyrosine-protein kinase Mer; IRF5, interferon regulatory factor 5; BCA-1, B-cell-attracting chemokine; BCDF, B-cell differentiation factor; TLR, toll-like receptor; RANK, receptor activator of nuclear factor kappa-B; RANKL, receptor activator of nuclear factor kappa-B ligand; EGF, epidermal growth factor; EGFR, epidermal growth factor receptor; PG, prostaglandin.

SMs, which are immune cells, are involved in the pathogenesis of a variety of inflammatory arthritis conditions. In light of the previously unclear classification of the SM subpopulations, little was known about whether such cells had protective or destructive functions during disease. In order to visualize SMs and study their spatial and temporal distribution at steady state, as well as during arthritis, Culemann et al. (21) labeled the chemokine receptor CX3CR1 and combined it with fluorescence microscopy to follow SMs in mice. In doing so, CX3CR1^-^ interstitial macrophages were shown to appear as self-renewing precursors of CX3CR1^+^ SMs, and that their emergence appeared earlier than the formation of immune complexes due to immune infiltration. These SMs are membranous structures capable of expressing polarity-related molecules or scavenger receptors, providing an anti-inflammatory barrier to the joint. Alivernini et al. (22) also confirmed that in human synovial tissue, the expression of MerTK distinguished SMs with a protective phenotype (MerTK^+^) from those with a pro-inflammatory phenotype (MerTK^-^). These MerTK^+^ cells exhibited different regulatory characteristics depending on the disease state (healthy, active, or in remission), and were able to secrete tight junctional proteins similar to epithelial cells, which hindered the transport of immune cells in a steady state. In addition, protective SMs could express high levels of anti-inflammatory mediators, such as IL-1 receptor antagonist (IL-1RA) or osteoprotegerin (OPG), and may act acted as negative regulators of pro-inflammatory cytokines, effectively preventing inflammatory cell infiltration as well as associated bone destruction (5). SMs have also been shown to secrete TNF-α, IL-6, IL-23, and large amounts of CXCL together with CCL chemokines to promote and maintain inflammation through the recruitment and activation of polymorphocytes (PMNs), T cells, B cells, or monocytes (78). These specific types of SMs and levels of inflammatory progression have been shown to form a positive feedback loop that accelerates the rate of inflammation development. According to the dual role of SMs in joint inflammation, a better understanding of the link between this cell and other immune cells may help to more accurately characterize their pathogenic function (**Figure 2**), thus fostering the development of SM-targeting strategies.

### SMs and Lymphocytes

Synovial tissue in patients with arthritis can be considered as tertiary lymphoid tissue or ectopic lymphoid structures, which are similar in structure to secondary lymphoid tissue, with the presence of T-cell and B-cell differentiation sites (79). SMs as a type of macrophage may also drive T lymphocyte infiltration, triggering B lymphocytes to produce immunoglobulins and further aggravate the inflammatory response.

It was found that an increase in CXCL16^+^ SMs in RA synovium led to the recruitment of CXCR6^+^ memory T cells, which, in turn, triggered the inflammatory cascade response associated with RA pathology (80). van Roon et al. (81) used a novel drug-coupled antibody (CD64-CaMi) against the IgG high-affinity receptor (FcgammaRI) to culture monocytes in the peripheral blood and synovial fluid of RA patients, where CD64-CaMi was shown to induce SM death along with the effective inhibition of pro-inflammatory Th1 cytokine production. Their findings suggested that the SMs played an important part in activating inflammation-promoting autoreactive T cells, thus triggering and exacerbating the disease. Moreover, inflammatory macrophages have been observed to express IRF5 so as to promote the proliferation and activation of T lymphocytes by secreting IL-12, IL-23, and IL-1β, while promoting the transformation of T lymphocytes to the Th1 or Th17 phenotype (82). If the IRF5–IRF4 modulation axis is designated as a new target for therapeutic intervention, inhibition of IRF5 activity in SMs may specifically affect the expression of pro-inflammatory cytokines and lead to a decrease in effector T cells. B cells are essential antigen precursor cells in proteoglycan-induced arthritis (PGIA). The presence of MerTK^-^HLA^high^CD48^+^ SMs in healthy and arthritic synovium may be key at the onset of inflammation (22). Previous studies have detected B-cell-attracting chemokines (BCA-1) (83) and B-cell differentiation factor (BCDF) (84), which have been shown to be potent pro-B-cell aggregation factors in the condition medium of synovial cells in patients with inflammatory arthritis. B cells have also been noted to secrete IL-1, IL-6, TNF-α, and IFN-γ, which can directly or indirectly stimulate macrophages in the synovial lining and sub-synovial layer, further leading to the destruction of cartilage and bone (85). Therefore, anti-CD20 antibody therapy may affect both mature B lymphocytes and SMs. Comparative transcriptomic analysis has also revealed that gene patterns of activated B cells and T cells in RA synovial tissue reflects associated response to activated macrophages (37).

### SMs and SFs

Synovial tissues are immune networks that have complex components. Through the use of fluorescence tracking of SMs in arthritis mouse models after the induction of synovitis, interactions between SFs and SMs in the lining layer were found to occur due to active remodeling, resulting in the loosening of barrier layers (21). Therefore, whether the abnormal activation and proliferation of SMs and SFs in arthritis patients are related to their interaction has continued to be the focus of research.

Kuo et al. (38) found that long-term exposure to pro-inflammatory environments could lead to the production of prostaglandins by SFs together with inflammatory factors, thereby prompting SMs to enter a state different from classical M1 and M2 polarization. Meanwhile, these macrophages have been shown to promote the invasion of fibroblasts in an epidermal growth factor receptor-dependent manner. The associated findings suggest that intercellular crosstalk in the inflammatory environment remodels both cell types and accelerates their mediated joint destruction. The team then co-cultured HBEGF^+^ SMs with SFs, in which the RNA sequence analysis revealed that 855 SF genes, including IL-11, IL-33, and IL-6, were altered. Furthermore, inflammation–macrophages–fibroblasts were shown to form an interacting system, in which targeting HBEGF^+^ SMs may serve as a new therapeutic pathway in order to alleviate inflammation. In addition, an analysis of SMs that adopted single-cell sequencing techniques showed that MerTK^+^CD206^+^ SM clusters could induce inflammatory responses to SFs and promote synovial inflammation through the production of pro-inflammatory cytokines in conjunction with alarm proteins. In contrast, MerTK^-^CD206^-^ SMs clusters were shown to produce lipid mediators that resulted in the induction of a repair response to SFs as well as a reduction in synovial inflammation. Meanwhile, SFs have been noted to promote the receptor activator of nuclear factor kappa B ligand (RANKL) production by macrophages, induce OC formation, and exacerbate bone destruction (86).

### SMs and OCs

OCs are multinucleated giant cells that promote bone resorption; their epigenetic and transcriptional changes are mainly dependent on macrophage colony-stimulating factor (M-CSF) and RANKL (87). Macrophages have been found to differentiate directly into mature OCs under specific microenvironmental conditions (88). Mature OCs have also been shown to be involved in pathological bone erosion in patients with arthritis, which occurs when the pannus invades the outer surface of articular bone. Accordingly, whether this process involves SMs has always been a question of inquiry.

Cuda et al. (89) found that high activation of SMs in patients with arthritis increased the expression of toll-like receptors (TLR2, TLR3, TLR4, and TLR7) and produced a large number of pro-inflammatory cytokines (IFN-γ, TNF-α, IL-1, and IL-6), chemokines (CCL5, CXCL1, and CXCL10), and various matrix lyases, which, in turn, activated OCs to promote bone destruction. Additionally, the mannose incorporated liposomal delivery system (ML) delivers p-coumaric acid (CA), a dietary polyphenol, to SMs of adjuvant-induced arthritis (AIA) rats that inhibit OC differentiation and bone resorption (90). Notably, scientists have discovered a group of arthritis-associated osteoclastic macrophages (AtoMs) (CX3CR1^hi^Ly6C^int^F4/80^+^I-A^+^/IE^+^ macrophages) in the synovial tissue of mice suffering from collagen-induced arthritis, which has been shown to be capable of differentiating into functional osteoclastic precursors in the pannus (91). Similar cell populations (CX3CR1^+^ HLA^-^DR^hi^CD11c^+^CD86^+^ SMs) have also been noted in the synovial tissues of patients with RA. When these AToMs are inhibited by thiostrepton, osteoclastogenesis can be inhibited simultaneously, serving as a potential target for RA treatment.

## Research Progress of SMs in Inflammatory Arthritis

### Research Progress of SMs in the Treatment of RA

As research continues to develop, the importance of SMs in the pathogenesis of RA is progressively being studied. SMs have been shown to secrete a variety of cytokines and chemokines while regulating proliferation *via* signals such as transcription factors. In recent years, treatment options for RA have expanded, which are now composed of corticosteroids, traditional synthetic DMARDs (CsDMARDs), biological DMARDs (BDMARDs), and targeted synthetic DMARDs (TsDMARDs) (92). These treatment modalities involve multiple mechanisms of action and have direct or indirect effects on the treatment of SMs. Therefore, the targeted intervention of SMs may have potential applications in the treatment of arthritis (**Table 3**).

**Table 3:** Commonly used drugs for the treatment of inflammatory arthritis by SMs.

Disease	Type of drug	Name of drug	Mechanism of action	Reference
RA	Medicinal herb	Sinomenine (SIN)	Reduce the number of pro-inflammatory SMs in synovial tissue	Liu et al. (39)
	NF-κB inhibitors	Withaferin-A	Promotes SMs (CD11b^+^) repolarization	Sultana et al. (40)
	Medicinal herb	Celastrol	Consumption of pro-inflammatory CD68^+^SMs	Cascão et al. (41)
	Compound	Methyl palmitate	Consumption of pro-inflammatory CD68^+^SMs	Abdel Jaleel et al. (42)
	Compound	Cilostazol	Attenuates the expression of IL-23 co-localized with CD68^+^SMs in the synovium	Park et al. (43)
	Immunomodulators	Methotrexate (MTX)	Reduce the number of activated macrophages	Gremese et al. (44)
	JAK inhibitors	Tofacitinib ruxolitinib	Inhibited the stimulation of TNF and the activation of STAT signaling pathways in SMs; reduced the nuclear localization of NF-κB subunits in SMs	Yarilina et al. (45)
	Biologics	Infliximab	Reduces Ly6C macrophage infiltration in pannus	Huang et al. (46)
	Biologics	Rituximab	Reduce TNF and IL-6 in the microenvironment, indirectly affecting SM activation	Teng et al. (47)
	Biologics	CTLA4-Ig	Downregulated T-cell activation and SMs secreted IL-6, TNF-α, and IL-1β	Brizzolara et al. (48)
OA	compound	Quercetin	Induction of polarization of M2-type SMs	Hu et al. (49)
	Nonsteroidal anti-inflammatory drugs	Diclofenac sodium	Induction of polarization of M2-type SMs	Xin et al. (50)
	Medicinal herb	The anti-swelling formula of Fangji Huangqi	Suppresses polarization of M1-type SMs	Wei et al. (51)
	Compound	Itaconate	Regulate the polarization state of the SMs; directly or indirectly inhibits inflammation and senescence of chondrocytes	Ni et al. (52)
	Synthetic nanoparticles	Modified zeolitic imidazolate framework-8 (ZIF-8) nanoparticles	Facilitates the change of the polarization state of SMs from the M1 phenotype to the M2 phenotype	Zhou et al. (53)
PsA	Glycolytic inhibitors	2-DG/HIF1αi	Reverses the metabolic reprogramming and expression of IL-1β, IL-6, and IL-12 in SMs	Van Raemdonck et al. (54)
	Monomeric glycoproteins	GM-CSF	Drive a change in the polarization state of the M1-type SMs	Fuentelsaz-Romero et al. (55)

Other targeted intervention SMs for the treatment of inflammatory arthritis are not listed in **Table 3**, such as gene editing and external forces.

Sinomenine is an active monomer obtained from the Chinese herbal medicine Qingteng, which has been shown to reduce the number of pro-inflammatory SMs in synovial tissue and improves arthritic symptoms in RA mice (39). Withaferin-A, a steroidal endoester-encapsulated mannose modified with liposomes, can be used to improve RA by promoting SMs (CD11b^+^) repolarization in AIA rats (40). CD68^+^ SMs act as pro-inflammatory macrophages that accelerate the onset of arthritis. Therefore, targeted intervention of this subset of cells may effectively alleviate the pathogenesis of RA. Moreover, depletion of SMs (CD68^+^) with clodronate-containing liposomes has been found to inhibit the onset and development of antigen-induced arthritis models (93). Rita et al. (41) injected celastrol that was isolated from the Chinese herbal medicine triptolide into the tail vein of AIA rats, in which a significant reduction in the number of CD68^+^ SMs and overall synovial inflammatory cells were observed 22 days later, thus preventing joint destruction without side effects. Methyl palmitate has been shown to inhibit the expression of CD68^+^ SMs in adjuvant-induced rat models of arthritis and can exert potential anti-inflammatory effects (42). Cilostazol has also been shown to significantly attenuate the expression of IL-23 co-localized with CD68^+^ macrophages in the knee synovium of CIA mice through cAMP-dependent protein kinase activation while reducing the severity of arthritis (43). MTX reduces the presence of activated macrophages in the joints, liver, and spleen of arthritic rats and has been widely used in the treatment of RA (44).

Currently, targeted interventions that can modulate factors and microRNAs associated with the phenotypic transformation of SMs have also garnered increased attention. Specifically, GM-CSF phase II randomized controlled trials have demonstrated that GM-CSF inhibitors for RA have a high safety profile as well as a very low chance of infection complications (94). miR-155 has been detected in the BIC gene on mouse chromosome 16 and human chromosome 21. Moreover, studies in clinical and animal models have revealed that miR-155 is associated with RA pathogenesis, which can mediate the upregulation of SM expression in patients with RA (95). CRISPR/CAS9 technology has also been used to genetically edit mouse macrophages, while further analysis showed that this technology can reduce pro-inflammatory cytokines produced by macrophages by targeting NLRP3 inflammatory bodies, which may serve as a target in improving inflammatory diseases (96). Jia Xu et al. (97) used bioinformatics to systematically analyze the GSE97779 and GSE10500 expression profiles of SMs in RA patients, identifying 10 candidate genes (FN1, VEGFA, HGF, SERPINA1, MMP9, PPBP, CD44, FPR2, IGF1, and ITGAM) that may be used in the future diagnosis, prognosis, and treatment of RA.

JAK inhibitors have long been developed as anti-inflammatory and immunosuppressive agents, of which tofacitinib and ruxolitinib have exhibited a significant degree of clinical efficacy in RA. Yarilina et al. (45) investigated the mechanism of action of JAK inhibitors, in which the stimulation of TNF, the activation and expression of STAT-1, and downstream inflammatory target genes in RA SMs were inhibited. In the interim, JAK inhibitors can also reduce the nuclear localization of NF-κB subunits in SMs. In this regard, targeted SMs have been shown to play an important role in the treatment of RA joint inflammation. In addition, targeting SMs by TNF-α inhibitors (98), Bruton’s tyrosine kinase (BTK) inhibitors (99), and sex hormone modulators (100), and clearance of overexpressed IgG high-affinity receptors (FcγRI) (81) can be useful in the treatment of RA. Symptomatic improvement of patients with arthritis following infliximab therapy have been shown to be accompanied by a significant decrease in infiltration of Ly6C macrophages in the pannus (46). Although rituximab is an anti-CD20 antibody that acts against B cells, it may indirectly affect SM activation by reducing the production of TNF and IL-6 in the microenvironment (47). Similarly, cytotoxic T lymphocyte-associated antigen 4 immunoglobulin (CTLA4-Ig) can bind to B7 molecules on antigen-presenting cells in order to downregulate T-cell activation. Co-culture of the biologic agent CTLA4-Ig with SMs from RA patients has also been shown to significantly downregulate the expression of cytokines IL-6, TNF-α, and IL-1β, indicating that CTLA4-Ig has an indirect and direct anti-inflammatory effect on primary monocultures of RA SMs (48). Cholesterol-activated liver X receptors (LXRs) are highly upregulated in RA SMs and can enhance TLR-driven cytokine release, such as TNF-α (101). In addition, MerTK^+^CD206^+^ SMs appear to play a crucial role in maintaining the sustained remission of RA inflammation. A decrease in the proportion of MerTK^+^ SMs during remission has been found to be associated with an increased risk of disease after drug discontinuation. Therefore, the regulation of MerTK^+^ SMs may be a potential therapeutic approach for RA (22). Importantly, several new techniques, such as the use of positron emission tomography (PET) scanning and activation of SMs-induced tracer-targeting molecules, may help improve the effectiveness of targeted therapies for SM-mediated arthritis (19).

### Research Progress of SMs in the Treatment of OA

OA has long been recognized as a degenerative disease of cartilage that may be accompanied by secondary bone injury and osteophytes (102). Mild synovial inflammation, which is a combination of macrophage-based inflammatory infiltrates, has been observed in more than half of OA patients in both the early and late stages of disease. Although inflammation is less pronounced, ample evidence has been produced to support its pathogenic role (103). Currently, the importance of synovial membranes, especially SMs, in OA has been elaborated in both *in vitro* and *in vivo* studies.

Yarnall’s team studied synovial tissue from experimental dogs with cruciate ligament rupture (CR) and OA. Here, an increased number of CD68^+^, CCR7^+^, and iNOS^+^ cells in the CR and OA groups was seen compared to that of the normal group, suggesting that these M1-type pro-inflammatory SMs played considerable roles in their pathogenesis. Their findings also provided evidence that these cells may serve as therapeutic targets in their treatment (104). Moriya et al. (105) examined synovial tissue collected from knee joints of OA patients, and found that SM-induced C-type lectins (Mincle) may play a significant role in synovial inflammation of OA patients, which may be potentially developed into a new target for OA therapy. SMs have been reported to respond to danger-related molecular patterns, including cartilage fragments and intracellular proteins in necrotic cells (106). During the same period, rheumatologists from six academic institutions in the United States biopsied the synovial tissue of RA and OA patients *via* minimally invasive ultrasound, and found a high degree of transcriptional heterogeneity (107), indicating that multiple subsets of SMs may exist in the same joint. The persistence of different subpopulations of SMs in inflammatory disease may contribute to OA treatment. Specifically, an analysis of patients with knee OA based on the gene expression profiles of SMs showed a rise in number and tight alignment of synovial CD14^+^ macrophages, which exhibited characteristics of cell proliferation as well as a high expression of Ki67 (108). Moreover, *in vitro* studies have demonstrated that the depletion of CD14^+^ macrophages in synovial cell cultures can lead to a reduction in IL-1β, TNF-α, MMPs, and Aggrecanase enzymes capable of degrading articular cartilage (109). Recently, Thomson et al. (110) showed that key OA mediators (TNF, IL-6, and IL-1β) were released into the joint space *via* HLA-DRA^+^ macrophages and neutrophils (NE), after which it again showed that tissue-specific targeting of synovial pathogenic molecules or cells has the potential to treat OA.

Studies involving human and animal models have shown that macrophages accumulated in synovial tissue are associated with pain sensitivity in OA joints (111). Sakurai et al. (112) reported that SMs were involved in pain in patients with advanced knee OA resistant to COX inhibitors by increasing pro-inflammatory mediators and that drugs targeting SMs may have beneficial analgesic effects. As modulators and producers of nerve growth factor (NGF) in joint synovial tissue, the role of SMs is regulated by TNF-α, which can treat joint pain in OA by upregulating the NGF signal transduction produced by SMs in OA joints (113). Abnormal mechanical stress exacerbates the pyroptosis of SMs through mechanically sensitive channel proteins, which can also provide pain relief in OA patients (114).

Intra-articular injection of oxidized low-density lipoprotein (ox LDL) into the knee joint of mice that acts on SMs has been shown to promote transforming growth factor β (TGF-β) signal transduction and prevent the influx of S100A8/S100A9-producing cells, thereby inhibiting joint inflammation (115). In addition, genes such as miR-9-5p (116), miR-492 (117), miR-92a-3p (118), miR-135b (119), and miR-155 (120) have been observed to regulate the polarization state of SMs, inhibiting the progression of OA. Quercetin induces the polarization of M2 SMs and upregulates the expression of TGF-β along with insulin-like growth factor (IGF), which then creates a cartilage-promoting microenvironment for chondrocytes in order to enhance cartilage repair in OA and exert cartilage protective effects (49). Diclofenac sodium similarly establishes an anti-inflammatory microenvironment by promoting the polarization of M2 SMs, attenuating pain and cartilage degeneration in maternal immune activation (MIA)-induced OA rats (50). The anti-swelling formula of Fangji Huangqi has also been described to improve joint synovitis in OA rats *via* inhibition of M1 SM polarization and reduction in the secretion of both pro-inflammatory cytokines and metal-matrix proteases (51). Meanwhile, exogenous supplementation of Itaconate has been shown to improve OA progression by regulating the polarization state of SMs and directly or indirectly inhibiting inflammation and the senescence of chondrocytes, making it a potential drug for the treatment of OA (52). Zhou et al. (53) synthesized modified zeolitic imidazolate framework-8 (ZIF-8) nanoparticles (NPs) by regulating intracellular gases and reprogramming metabolic phenotypes, which has been shown to polarize macrophages in synovial tissue from pro-inflammatory M1 phenotypes to anti-inflammatory M2 phenotypes, a strategy that may offer novel approaches for OA treatment. In order to identify more therapeutic targets, further research into the role of SMs in OA does face some exciting moments.

### Research Progress of SMs in the Treatment of PsA

Psoriatic arthritis (PsA) is a chronic, immune-mediated inflammatory arthropathy with lesions that mainly involve attachment points and tendon sheaths. Although synovial hyperplasia is not obvious, histopathological examination of the synovium has exhibited hyperplasia of the lining layer with more SMs being visible (121, 122). Therefore, synovial cells such as SMs are thought to play an essential part in inducing inflammation and destruction of PsA joint tissue and skin.

Compared to other patients with inflammatory arthritis, patients with PsA have more aggressive inflammation of synovial tissue, which is primarily driven by T cells and causes hyperproliferation of synovial lining cells (123). Hornum et al. (124) confirmed *via* double immunostaining of C5aR and CD68 that C5aR^+^ cells in the synovium of PsA and RA are predominantly macrophages. Moreover, C5aR^+^ cells have been shown to be closely related to T cells, whose interrelationship may likely play a pathogenic role. Further studies have also revealed that therapeutic targeting of the C5a–C5aR axis could effectively inhibit the proliferation of SMs in PsA patients, thus reducing synovial inflammation. Meanwhile, Tang et al. (125, 126) demonstrated that prolactin receptors (PRLRs) are predominantly present on SMs in patients with RA and PsA through mRNA sequencing, in which SMs with INF-γ and IL-10 polarization were observed to express the highest PRLR values. The authors speculated that when PRL activates PRLR in SMs, it induces the production of pro-inflammatory cytokines. Therefore, targeted intervention of prolactin sites in inflammatory cells may serve as a novel form of treatment for arthritis. Nicotinic alpha 7 acetylcholine receptor (α7nAChR) is present in SMs and SFs of RA patients and PsA patients as a regulatory mediator for specific cholinergic anti-inflammatory pathways, and inhibition of the expression of this receptor may slow down the progression of the disease (127). The endogenous TLR7 ligands miR-29 and miR-Let7b have been shown to be significantly increased in the synovial fluid of PsA patients compared to that of OA patients. Intradermal (id) injection of miR-let7b can amplify Th1 cells and CD68^+^ M1 SMs, upregulating the transcription of glycolytic mediators GLUT1, C-MYC, and HIF1α. In addition, it has been shown to exacerbate skin inflammation, suboptimal joint inflammation, and metabolic remodeling of PsA-like preclinical models. Thus, glycolytic inhibitors may act on SMs and reverse skin-joint crosstalk in PsA (54). Compared with persistent undifferentiated arthritis (UA), the density of CD163^+^SMs has been noted to be significantly increased when UA evolves into PsA (UA-PsA). Furthermore, during this phase, GM-CSF drove alterations in the polarization state of pro-inflammatory SMs. Therefore, it is reasonable to posit that GM-CSF may serve as a potential therapeutic target for SMs in UA-PsA (55). Pawel et al. (128) found a myeloid Tie2 signal in PsA synovial tissue, the participation of which has been described to be sufficient and necessary in promoting synovial inflammation in PsA. Tie2 and inflammatory signaling pathways can synergistically regulate the ability of SMs in expressing inflammatory genes. Therefore, targeting both pathways simultaneously may confer a therapeutic effect on PsA.

Overall, research pertaining to SMs for the treatment of PsA primarily seeks to decrease anti-inflammatory SMs while preventing the excessive infiltration of pro-inflammatory SMs in the synovium. Since cell surface markers do not specifically label SMs, the role of endogenous environmental factors and related changes in hormones and cytokines affect the distribution and response of cells *in vivo*. Great challenges continue to exist in the treatment of PsA for different SM subpopulations. Therefore, conducting further detailed studies on the identification of different SM subtypes and their regulation while formulating therapeutic strategies based on their pro-inflammatory and anti-inflammatory properties to search for novel targets with high specificity, high sensitivity, and low side effects should be prioritized.

## Conclusion

SMs, as one of the major cell types that constitute the synovium of the joint, play an important role in the pathogenesis of inflammatory arthritis due to their immunomodulatory functions in different activation states. With the continuous deepening of research, a variety of targeted drugs for SMs have entered preclinical treatment studies. Researchers try to find drugs that specifically target different subpopulations of SMs, with a view to acting on both pro-inflammatory SMs and anti-inflammatory SMs. However, the pathogenesis of arthritis is complex, the relevant regulatory factors and modulators of SMs in different origins have yet to be identified, and the interactions between the various cell subtypes remain unclear. Therefore, compared with traditional treatment methods, the safety and efficacy of SM-targeted therapy must be further investigated.

## Data Availability Statement

The original contributions presented in the study are included in the article/supplementary material. Further inquiries can be directed to the corresponding author.

## Author Contributions

This article is mainly written by L-KB. Y-ZS and X-XW wrote part of the manuscript and proofread the manuscript. BB and C-QZ helped us collect literature information and draw pictures. G-LZ and L-YZ reviewed the manuscript and proposed final revisions. All authors contributed to the article and approved the submitted version.

## Funding

This work was supported by the Shanxi Province Overseas Students Science and Technology Activities Merit-based Funding Project [grant number 20210003].

## Conflict of Interest

The authors declare that the research was conducted in the absence of any commercial or financial relationships that could be construed as a potential conflict of interest.

## Publisher’s Note

All claims expressed in this article are solely those of the authors and do not necessarily represent those of their affiliated organizations, or those of the publisher, the editors and the reviewers. Any product that may be evaluated in this article, or claim that may be made by its manufacturer, is not guaranteed or endorsed by the publisher.

## Abbreviations

TRMs, tissue-resident macrophages; YSMPs, yolk sac-derived myeloid-biased progenitors; BMSMs, bone marrow-derived synovial macrophages; ESMs, embryonic synovial macrophages; c-FLIP/Flip, cellular-FLICE inhibitory protein; PGIA, proteoglycans induced arthritis; BCA-1, B-cell-attracting chemokines; BCDF, B-cell differentiation factor; AIA, adjuvant-induced arthritis; AtoMs, arthritis-associated osteoclastic macrophages; CR, cruciate ligament rupture; MIA, maternal immune activation; UA, undifferentiated arthritis; UA-PsA, UA evolved into PsA.
